# The crosstalk of caveolin-1 and autophagy in different diseases

**DOI:** 10.3389/fimmu.2025.1648757

**Published:** 2025-09-15

**Authors:** Yuting Yang, Qiqi Ma, Mei Yang, Ruixue Wei, Zhiguo Wang, Chunmeng Jiang, Hui Liu, Mei Han

**Affiliations:** ^1^ Department of Gastroenterology, Second Hospital of Dalian Medical University, Dalian, Liaoning, China; ^2^ Departments of Gastroenterology, Wuxi People’s Hospital, Wuxi Medical Center, The Affiliated Wuxi People’s Hospital of Nanjing Medical University, Nanjing Medical University, Wuxi, Jiangsu, China; ^3^ Intensive Care Unit (ICU), Diamond Bay District, The Second Affiliated Hospital of Dalian Medical University, Dalian, Liaoning, China; ^4^ Department of Gastroenterology, Air Force Medical Center, Beijing, China

**Keywords:** caveolin-1, autophagy, disease mechanisms, molecular regulation, signaling pathways

## Abstract

Caveolin-1 (Cav-1) is an important structural protein that constitutes the caveolae on the cell membrane. Cav-1 is expressed in various cells, especially in white adipocytes and endothelial cells. Cav-1 plays an important physiological role in regulating substance transport, signal transduction, and multiple metabolic pathways in the body. Autophagy degrades damaged organelles within cells and recycles them, thus playing an important role in maintaining homeostasis of the internal environment. Previous studies have found that Cav-1 is involved in the occurrence and development of multiple systemic diseases by regulating autophagy. In addition, autophagy can also affect the expression level of Cav-1 by degrading it. Therefore, there is a close regulatory relationship between Cav-1 and autophagy. Based on recent research progress, this article provides a detailed overview of the importance of the crosstalk between Cav-1 and autophagy in various systemic diseases such as cardiovascular, respiratory, and digestive systems. It aims to provide a more comprehensive understanding of the interaction between Cav-1 and autophagy, in order to promote further research and achieve clinical applications as soon as possible.

## 1 Introduction

**Figure 1:**
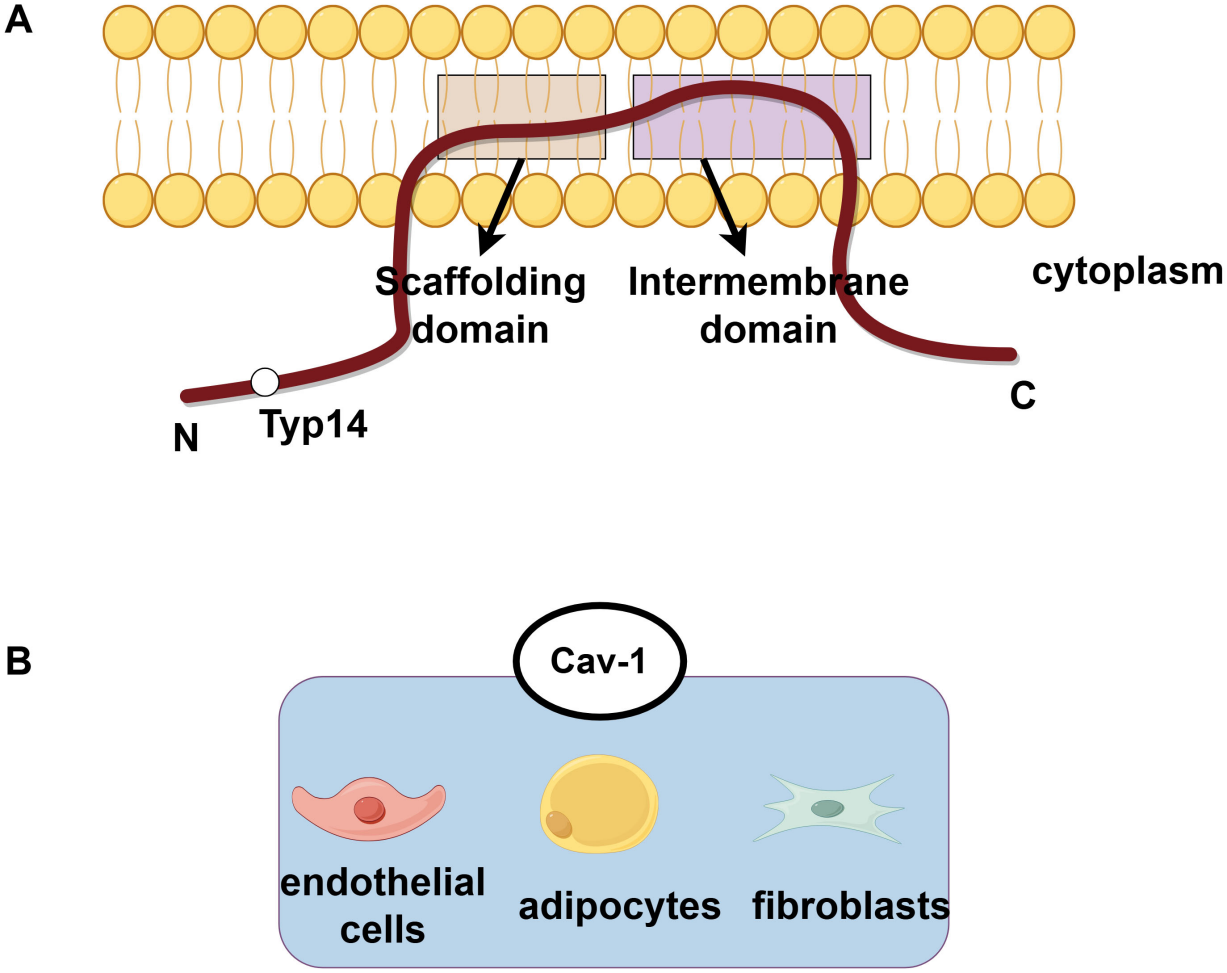
The protein structure of caveolin and the subcellular distribution of Cav-1. **(A)** The protein structure of caveolin exhibits a hairpin-like topology, with both its N-terminal and C-terminal ends facing the cytoplasm. Phosphorylation at the tyrosine-14 site is crucial for the function of Cav-1. **(B)** Cav-1 is highly expressed in adipocytes, endothelial cells, and fibroblasts.

Caveolin (Cav) is an important structural protein that constitutes the caveolae which is a 60-80nm wide invaginated pit on the cytoplasmic membrane. The caveolin family comprises three members (Cav-1, Cav-2, Cav-3). Structurally, all caveolins adopt a hairpin-like topology, and all isoforms contain the core structural domains: an “N-terminal oligomerization domain (residues 1-101)”, a “scaffolding domain (residues 82-101)”, and a “C-terminal transmembrane domain (residues 135-178)”. Cav1 and Cav2 sequences are 60% homologous and mainly expressed in non-muscle tissue, while Cav3 is 65% homology to Cav1 and mainly expressed in striated muscle tissue (1–3). Cav1 is highly expressed in adipocytes, endothelial cells(ECs), and fibroblasts, where it coordinates the formation of fossa and the organization of lipid rafts, but shows low expression in lymphocytes, neurons, and liver cells, and it is almost undetectable in renal proximal tubular cells (4–7). The tyr14 of Cav-1 is its specific phosphorylation site, which plays an important role in cell signal transduction and function (such as autophagy and endocytosis), and this region is less characterized in Cav-2/Cav-3. In terms of Cav2, it relies on Cav-1 for stabilization. In the absence of Cav-1, Cav-2 cannot oligomerize and undergoes lysosomal degradation, leading to the collapse of caveolar structures (8). Cav-1 and Cav-2 are co-expressed in pulmonary ECs and form hetero-oligomers, synergistically regulating pulmonary surfactant secretion and vascular permeability. In addition, Cav3 deficiency causes muscular dystrophy but does not induce compensatory upregulation of Cav1. As mentioned above, this tissue-specific distribution determines their distinct functions (9, 10) (**Figure 1**). Among these three caveolin proteins, Cav-1 is the most extensively studied. Cav-1 is involved in various cellular processes such as endocytosis and transport, and also regulates processes such as autophagy, glucose and lipid metabolism, and calcium signaling transduction (3, 11). The abnormal expression of Cav-1 and dysfunction of Caveolae are closely related to various diseases, including fatty liver disease (12), muscular dystrophy (2), infection (13), osteoporosis (14), cancer (15), cardiovascular disease (16), neurological disease (17), etc. For example, high expression of Cav-1 affects the prognosis of breast cancer patients (18). The expression level of Cav-1 is positively correlated with the invasiveness of hepatocellular carcinoma (HCC), and knocking out Cav-1 can inhibit the epithelial mesenchymal transition (EMT) process through Wnt/β-catenin pathway, thereby inhibiting the malignant behavior of HCC metastasis (19).

Autophagy is a metabolic process that plays an important role in maintaining cellular homeostasis, regulating nutrient metabolism and energy production, and resisting internal environmental imbalances caused by diseases. Autophagy is a process of catabolism that plays an important role in regulating metabolism and maintaining internal environmental. Autophagy can be divided into three types: macroautophagy, microautophagy, and chaperone-mediated autophagy (CMA) (20, 21). The autophagy we usually called refers to macroautophagy, which involves damaged organelles or proteins in cells being wrapped by autophagosomes, degraded into smaller molecules in lysosomes, and then released into the cytoplasm. These small molecules are used for regenerative synthesis metabolism and energy production (22). Microautophagy refers to the process in which substrates are directly engulfed into lysosomes through the invagination of lysosomal membranes, and then degraded by lysosomal proteases (23). CMA is often associated with heat shock proteins (HSPs), and chaperones recognize specific amino acid sequences on proteins and internalize them into lysosomes for degradation (24). Autopahgy process involves complex signaling pathways and many regulatory targets. In previous studies, it has been found that the occurrence and development of many diseases are related to autophagy, including aging, cancer, neurodegenerative diseases, and other diseases (25–28). For example, previous studies have found that exercise and limiting calorie intake may prolong lifespan by activating autophagy, balancing protein synthesis and circulation within cells (29). Silencing cadherin-6 (CDH6) can inhibit EMT and invasiveness in thyroid cancer patients by promoting autophagy (30).

In recent years, multiple literatures have reported the crosstalk between Cav-1 and autophagy which has been proven to play a role in multiple systems such as cardiovascular, digestive, respiratory, urinary, mammary reproductive, and endocrine systems. These diseases share pathological hallmarks such as oxidative stress, metabolic dysregulation and inflammation, which are centrally modulated by Cav-1-autophagy interactions. In order to enable researchers to fully understand the research progress on the relationship between Cav-1 and autophagy, and to promote the clinical application and translation of research results, we provide a review of the impact of the interaction between Cav-1 and autophagy on the occurrence and development of various systemic diseases.

## 2 Cav-1 and autophagy in cardiovascular diseases

Researches have found that in cardiovascular diseases, the targets regulated by Cav-1 and autophagy are mainly ECs and cardiomyocytes. Cav-1 regulates autophagy in ECs, affecting oxidative stress, metabolism, inflammatory response levels, and deposition of low-density lipoprotein (LDL) beneath the endothelium, thereby affecting vascular function. Multiple studies have shown that the absence of Cav-1 can activate autophagy (31–34). After knocking down Cav-1, autophagy is activated, the oxidative stress level of vascular ECs increased, and cellular energy utilization was altered (31). For example, the glycolytic pathway takes precedence over the consumption of free fatty acids (31). In addition, research on human umbilical vein endothelial cells (HUVECs) has suggested that cadmium, as a heavy metal targeting ECs, promotes autophagy with low concentrations, thereby inducing endothelial cell dysfunction. This may be related to the severe interference of cadmium with caveolae in ECs and the reduction of Cav-1 levels (33). However, some contrary evidence suggests that the absence of Cav-1 and increased autophagy play a protective role in atherosclerosis. Xinbo Zhang et al. have found that Cav-1 deletion can reduce atherosclerosis by promoting endothelial autophagy flux, inhibiting inflammatory reaction and macrophage infiltration. The intrinsic mechanism may be that Cav-1 directly binds to the autophagosome components (e.g. autophagy-related protein 5 (ATG5)-ATG12 complex) and affects autophagosome formation by regulating its localization in lipid rafts. The absence of Cav-1 leads to the release of ATG5 from the Caveolae, the increase of autophagosome formation, and the increase of autophagy flux, thus reducing inflammation and atherosclerosis (32). Interestingly, the activation of autophagy also induces the redistribution of Cav-1 to the intracellular membrane compartment (32). Weike Liu et al. showed that low blood flow shear stress (SS) which often generated in the atherosclerotic plaque area and bifurcation area suppressed mitophagy, thereby resulting in mitophagy impairment and endothelial cell dysfunctions. Mechanistically, low SS elevated Cav-1 expression, which subsequently upregulated miR-7-5p and downregulated SQSTM1/p62, leading to impaired mitophagy in endothelial cells (28). Therefore, targeting the Cav-1/miR-7-5p/SQSTM1 axis may restore endothelial homeostasis and mitigate atherosclerosis (28). Moreover, Cav-1 also modulates LDL transcytosis via autophagy-dependent pathways, as evidenced by its role in high glucose-induced LDL deposition (6, 29). In the mouse diabetes model, it has been found that high glucose reduces the transfer of Sirtuin 6 (Sirt6) from the nucleus to the cytoplasm, leading to an increase in the acetylation level of Cav-1. Because microtubule-associated protein 1 light chain 3 (LC3B) cannot recognize acetylated Cav-1, the autophagic degradation of Cav-1 is reduced, thereby promoting LDL translocation. Therefore, the deposition of LDL under ECs accelerates the progression of atherosclerosis (35). In gestational diabetes mellitus (GDM), chronic hyperglycemia suppresses the AMPK-mTOR-PIK3C3 pathway, thereby inhibiting CAVIN1-LC3B-mediated autophagic degradation of Cav-1, leading to Cav-1 accumulation. Eventually, the accumulated Cav-1 is used to form more caveolae in the cell membrane, increasing the endocytic transport of LDL, thereby leading to an increase in lipid accumulation beneath the endothelial (11). When autophagy is activated, Cav-1-mediated endocytosis of LDL is inhibited, and lipid deposition is reduced. Interestingly, Cav1 exerts a dual effect on autophagy because it binds to LC3B through different domains under high glucose treatment. For example, autophagy is inhibited when LC3B interacts with Cav-1 scaffolding domain, while autophagy is activated when LC3B interacts with the intramembrane domain of Cav-1 (11).

**Figure 2:**
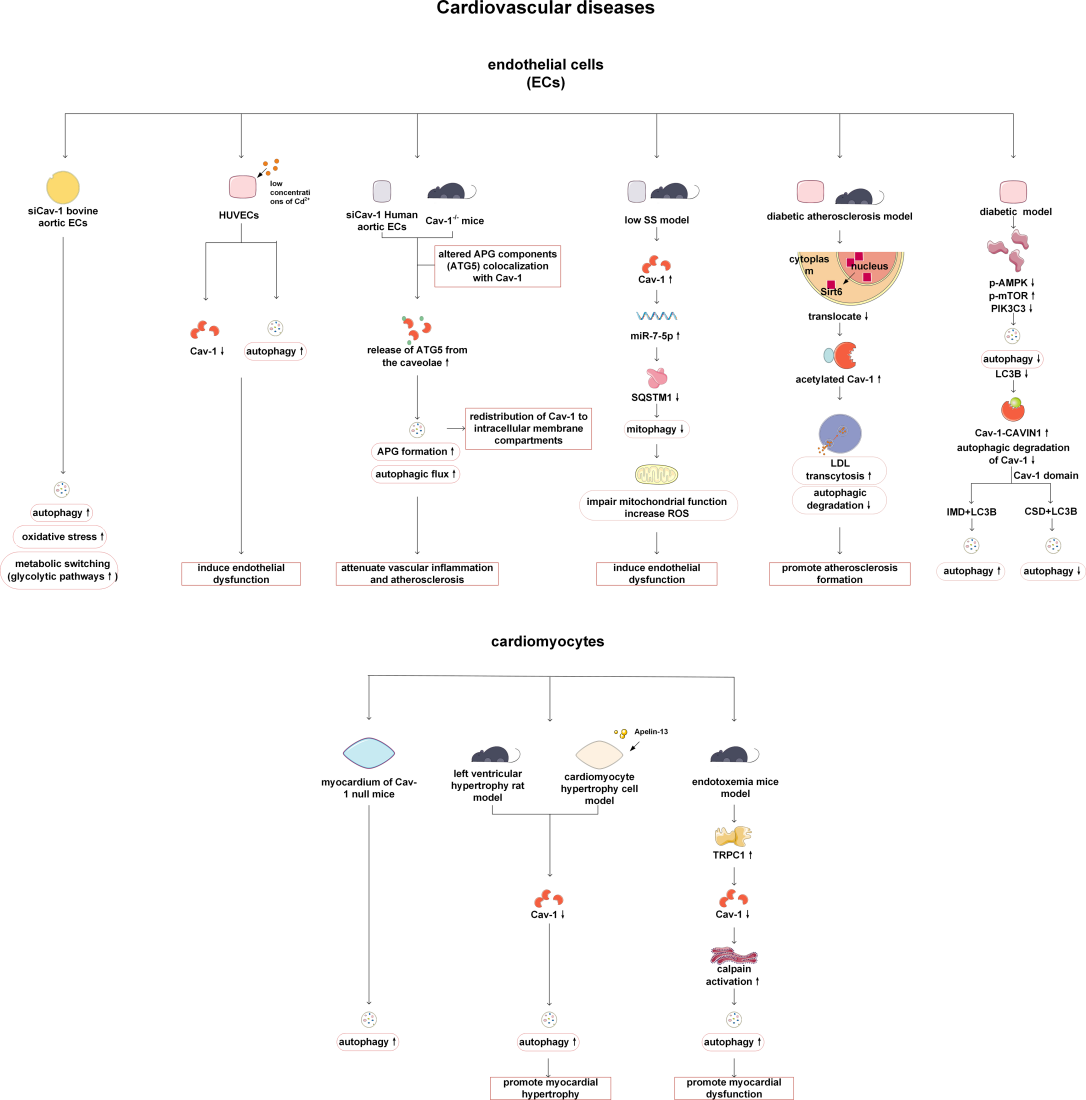
Cav-1 and autophagy in cardiovascular diseases. In endothelial cells, Cav-1 modulates autophagy, oxidative stress, and metabolic reprogramming. Cadmium may promote autophagy at low concentrations by severely interfering with caveolae and reducing Cav-1 levels. Deletion of Cav-1 reduces atherosclerosis and inflammation by promoting the release of ATG5 from caveolae and activating autophagy. SS impairs mitophagy through the Cav-1/miR-7-5p/SQSTM1 axis, leading to oxidative stress and endothelial dysfunction. Under high glucose conditions, the acetylation of Cav-1 (caused by less nuclear translocation of Sirt6) hinders the recognition of LC3B by Cav-1, inhibits autophagic degradation, promotes LDL transport and vascular lipid deposition. In GDM, suppressed AMPK-mTOR-PIK3C3 pathway inhibits CAVIN1-LC3B-mediated autophagic degradation of Cav-1. Notably, the interaction between different domains of Cav-1 and LC3B regulates autophagy in a bidirectional manner (inhibiting when bound to CSD and activating when bound to IMD). In cardiomyocytes, decreased expression of Cav-1 can activate autophagy and aggravate cardiac hypertrophy or cardiac dysfunction. Cav-1, caveolin-1; ECs, endothelial cells; ATG5, autophagy-related protein 5; HUVECs, human umbilical vein endothelial cells; APG, autophagosome; SS, shear stress; SQSTM1, sequestosome 1; ROS, reactive oxygen species; Sirt6, Sirtuin 6; LDL, low-density lipoprotein; AMPK, AMP-activated protein kinase; mTOR, mechanistic target of rapamycin kinase; PIK3C3, phosphatidylinositol 3-kinase catalytic subunit type 3; LC3B, microtubule-associated protein 1 light chain 3B; CAVIN1, caveolae associated protein 1; CSD, the CAV1 scaffolding domain; IMD, intramembrane domain.

In addition to acting on ECs, Cav-1 can also target cardiomyocytes and cardiomyoblasts to affect myocardial function by regulating autophagy. By observing the ultrastructure of cardiomyocytes from Cav-1 knockout mice, numerous large autophagosomes have been found in some cardiomyocytes which indicating increased autophagy in these cells (36). Cav-1 is considered as an important regulatory molecule for the development of myocardial hypertrophy. Cav-1 is reported to be downregulated in left ventricular hypertrophy (LVH) rat models and hypertrophic cardiomyocytes induced by apelin-13/apelin receptor (APJ), and autophagy pathway is also activated *in vitro*. When Cav-1 is overexpressed, autophagy is inhibited, and the diameter and volume of cardiomyocytes induced by apelin-13 are reduced, indicating that Cav-1 has a protective effect on cardiac hypertrophic disease (37). In mice with endotoxemia induced cardiac dysfunction, the decrease in Cav-1 expression mediated by Transient receptor potential canonical channel 1 (TRPC1) can activate autophagy in cardiomyocytes, which leading to impaired cardiac function (38). In a study of Icariin protecting rat heart-tissue derived embryonic cardiac myoblasts (H9c2 cells) from doxorubicin-induced cardiotoxicity, the expression levels of Cav-1, Beclin-1 and LC3II/LC3I were all deceased. Although no further experiments were conducted to verify whether Cav-1 directly regulates autophagy, it was demonstrated that the downregulation of Cav-1 has a protective effect. Further research is needed to determine whether Cav-1 plays a role in regulating autophagy during this process (39). In general, Cav-1 targets vascular ECs, cardiomyocytes and cardiomyoblasts, thus affecting the outcome of cardiovascular related diseases and providing a theoretical basis for clinical treatment (**Figure 2**).

## 3 Cav-1 and autophagy in respiratory diseases

In recent years, studies have found that Cav-1 plays an important role in the occurrence and development of respiratory diseases such as chronic obstructive pulmonary disease (COPD), hyperoxia induced lung injury, and pulmonary fibrosis by participating in autophagy of lung epithelial cells. Deletion of Cav-1 has been shown to increase starvation induced autophagy in Beas-2B cells, a type of human bronchial epithelial cell. The potential mechanism is that Cav-1 can bind to ATG5, ATG12, ATG12-ATG5 complex, and LC3B in Beas-2B, and regulate the expression of ATG16L. However, knockdown of Cav-1, starvation, and other stimuli can lead to dissociation with these complexes, and activated ATG12-ATG5 complex subsequently triggers autophagy, thereby aggravating lung epithelial damage (40). Autophagy is elevated in human COPD lungs as a response of cells and tissues to cigarette smoke (CS) exposure (41, 42). In the CS induced COPD mouse model, Cav-1 mediates the interaction between autophagy protein LC3B and external apoptosis factor Fas. Knockdown of Cav-1 makes epithelial cells sensitive to CS induced apoptosis. Cav-1 knockout mice exhibit higher levels of lung autophagy and apoptosis in response to chronic CS exposure *in vivo (* 43). Autophagy is activated in high oxygen induced lung injury. Cav-1 tyrosine 14 mediates the regulation of autophagy protein LC3B on the Fas apoptosis pathway, thereby reducing cell apoptosis and protecting lung epithelial cells. In summary, it can be found that Cav-1 plays an important role in lung injury by regulating the relationship between autophagy and cell apoptosis (44). Moreover, in animal and *in vitro* models of pulmonary fibrosis, it has been demonstrated that Cav-1-derived 7-mer peptide, Cav1 scaffolding domain peptide (CSP) 7, can prevent autophagy dysregulation by inhibiting p53 expression in alveolar-epithelial cells, and ultimately alleviate pulmonary fibrosis (45). However, Cav-1 has been found to have a negative effect in acute lung injury (ALI). In the lipopolysaccharide (LPS) induced mouse lung injury model, the level of Cav-1/nuclear factor kappa B (NF-κ B) axis is upregulated (46). Cav-1 knockdown *in vivo* and bone marrow-derived macrophages (BMDM) can activate autophagy by inhibiting protein kinase B (AKT)/mechanistic target of rapamycin (mTOR) and promoting adenosine 5’-monophosphate activated protein kinase (AMPK) signaling pathways, thereby alleviating the lung injury (46).

In the study of pulmonary arterial hypertension (PH), it has been found that Cav-1 can also participate in regulating autophagy of pulmonary vascular related cells. Lee SJ et al. have found that in PH, the expression levels of autophagy protein LC3B and its transcription factor Early growth response-1 (Egr-1) are increased in human pulmonary artery endothelial cells (PAEC) and human pulmonary artery smooth muscle cells (PASMC). Under normoxia, Cav-1 can colocalize with LC3B and Egr-1 on the cell membrane, while under hypoxia, Cav-1 can dissociate from LC3B and Egr-1, accompanied by activation of autophagy. This suggests that Cav-1 may be involved in the early stages of autophagy during PH (47).

In addition, Cav-1 can also act on lung cancer-related cells, altering tumor progression and patient prognosis by affecting autophagy processes. In cancer-associated fibroblasts (CAFs) of small cell lung cancer (SCLC), a decrease in Cav-1 expression can upregulate the transcriptional activation of its downstream molecule E2F transcription factor 1 (E2F1) and the expression of mitophagy marker BCL2 interacting protein 3 (BNIP3), leading to metabolic changes and inducing metabolic reprogramming pathways in tumor cells (48). In SCLC patients, the expression of the hedgehog (Hh) signaling pathway marker glioma associated oncogene-1 (Gli-1) has been found to be elevated, and negatively correlated with autophagy related marker-Cav-1 in CAFs. Therefore, the authors have inferred that Hh signaling pathway induces autophagy in the occurrence and metastasis of SCLC, thereby activating the tumor microenvironment in lung cancer cells. But in this article, Cav-1 is directly regarded as an autophagy related marker, and no deeper study has been done to prove the connection between Cav-1 and autophagy (49). In A549 (human non-small cell lung cancer, NSCLC) cells, treatment of resveratrol has been reported to increase autophagy, promote autophagy-mediated degradation of p62 and increase the formation of Fas/Cav-1 complex, thereby triggering activation of caspase-8 and cleavage of beclin-1 to initiate apoptosis. The lack of Cav-1 can prevent the formation of the Fas/Cav-1 complex to inhibit apoptosis and promote autophagy (50). Therefore, resveratrol plays a protective role in NSCLC by regulating Cav-1, autophagy, and apoptosis. However, more in-depth mechanisms such as how p62 regulates the formation of the Fas/Cav-1 complex need further studies to reveal (50).

**Figure 3:**
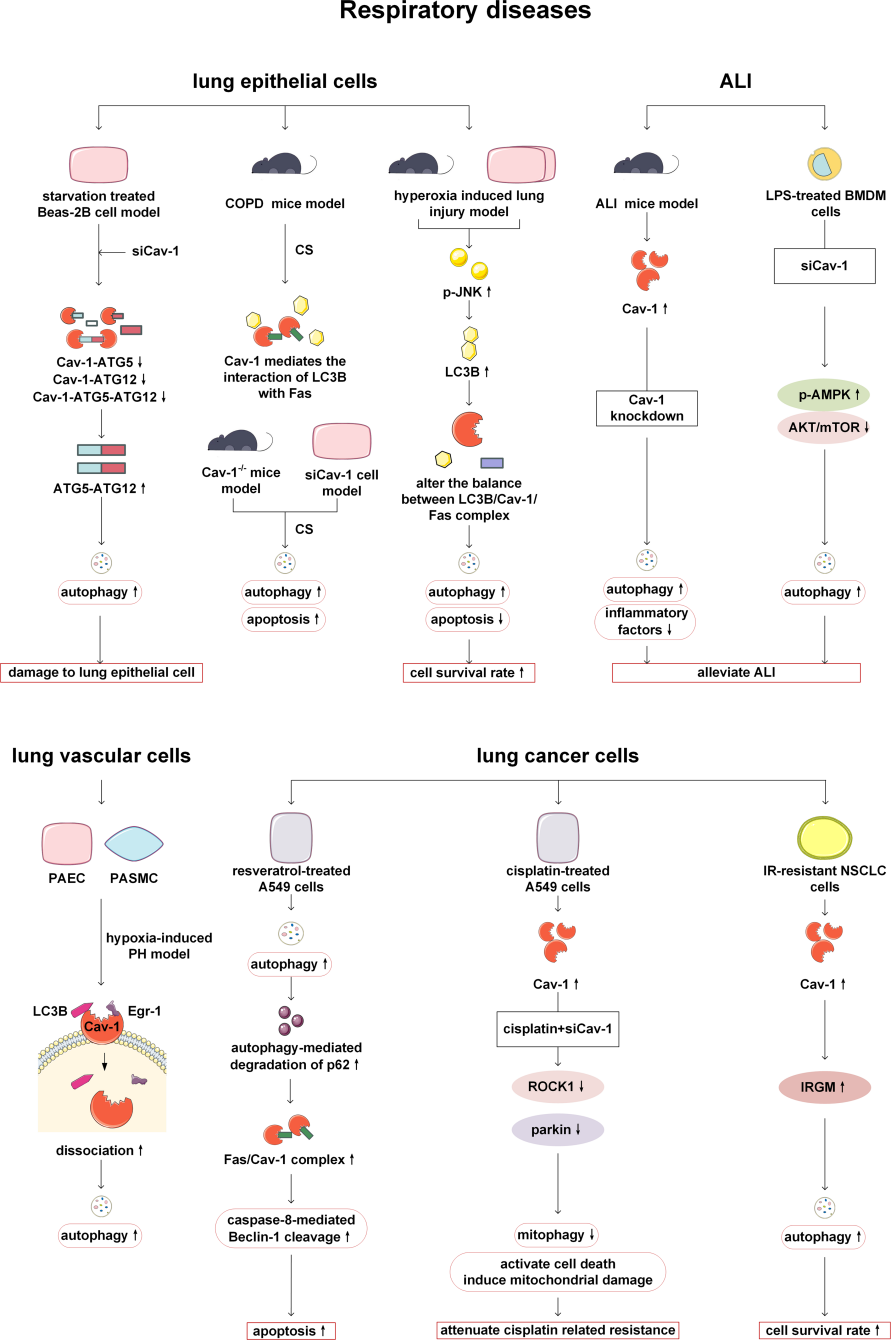
Cav-1 and autophagy in respiratory diseases. Knockdown of Cav-1 enhances the formation of the ATG12-ATG5 complex in lung epithelial cells, thereby promoting autophagy and leading to cellular damage. In COPD, Cav-1 modulates the equilibrium between apoptosis and autophagy by altering LC3B-Fas interactions. When the balance is disrupted, it leads to a simultaneous increase in autophagy and apoptosis, In contrast, in the hyperoxia-induced lung injury model, disruption of the Cav-1/Fas axis results in divergent changes in autophagy and apoptosis. During ALI, Cav-1 knockout enhances autophagy by inhibiting AKT/mTOR and activating the AMPK pathway, thereby alleviating inflammation. In pulmonary hypertension (PH), Cav-1 dissociation from LC3B/Egr-1 under hypoxic conditions triggers autophagy and exacerbates disease progression. In lung cancer, resveratrol promotes the formation of the Fas/Cav-1 complex, initiating apoptosis via autophagy activation. Furthermore, Cav-1 mediates resistance to cisplatin and radiotherapy by regulating the ROCK1/Parkin/mitophagy axis or IRGM/autophagy pathways, respectively; targeting these mechanisms can potentially reverse treatment resistance. ATG, autophagy-related protein; COPD, chronic obstructive pulmonary disease; CS, cigarette smoke; LC3B, microtubule-associated protein 1 light chain 3B; JNK, c-Jun N-terminal kinases; ALI, acute lung injury; LPS, lipopolysaccharide; AMPK, AMP-activated protein kinase; mTOR, mechanistic target of rapamycin kinase; AKT: protein kinase B; PAEC, pulmonary artery endothelial cells; PASMC, pulmonary artery smooth muscle cells; PH, pulmonary arterial hypertension; Egr-1: Early growth response-1; ROCK1, Rho-associated coiled-coil kinase 1; NSCLC, human non-small cell lung cancer; IR, irradiation-resistant; IRGM, immunity-related GTPase family M protein.

Cav-1 and autophagy are also involved in the process of chemotherapy and radiotherapy in lung cancer. In cisplatin-treated lung cancer cells, mitochondrial stress signaling has been induced, and excessive mitochondrial dysfunction triggers the activation of mitophagy, thereby attenuating the pro-apoptotic effect of cisplatin on A549 cells and leading to the occurrence of drug resistance. However, this process may involve the Cav-1/Rho-associated coiled-coil kinase 1 (ROCK1)/Parkin axis. The expression levels of Cav-1, ROCK1 and Parkin in lung cancer cells treated with cisplatin are increased, and Cav-1 knockdown can inhibit ROCK1, downregulate Parkin related mitophagy. Therefore, intervening in the Cav-1/ROCK1/Parkin axis can improve resistance to cisplatin in cancer treatment by inhibiting mitophagy (51). Radiotherapy plays an important role in the treatment of NSCLC, however, radioresistance is the main reason for the failure of treating NSCLC. In irradiation-resistant NSCLC parental cell lines, the expression of Cav-1 is increased. Overexpression of Cav-1 can promote autophagy by upregulating the expression of immunity-related GTPase family M protein (IRGM) to increase the survival of irradiation-resistant cells. Therefore, so inhibiting Cav-1 can improve the sensitivity of NSCLC radiotherapy (52). Taken together, Cav-1 and autophagy play an important regulatory role in a variety of respiratory diseases and are expected to be potential targets for future treatment (**Figure 3**).

## 4 Cav-1 and autophagy in digestive diseases

Previous studies have shown that Cav-1 can act on digestive organs such as liver, stomach and colorectum by regulating autophagy. The role of Cav-1 and autophagy in the liver is mainly reflected in reducing lipid accumulation and inflammatory response. Cav-1 expression is reduced in nonalcoholic fatty liver disease (NAFLD) mice and L02 cells treated with alcohol and oleic acid (A/O). Mechanistically, Cav-1 overexpression alleviates autophagy inhibition by suppressing Akt/mTOR signaling, and then degrades lipid droplets and reduces liver steatosis (53). Cav-1-regulated autophagy also plays a role in reducing lipid accumulation in acetaminophen aggravated alcoholic fatty liver disease. Cav-1 attenuates lipid accumulation by activating the PTEN-induced kinase 1 (Pink-1)/Parkin-related mitophagy pathway after acetaminophen and alcohol treatment in mice and L02 cells (54). In a mouse model of liver injury induced by LPS stimulation, superparamagnetic iron oxide nanoparticles (SPIONs) enhanced autophagy by activating the Cav-1-Notch1/HES1 signaling pathway, thereby upregulating the level of the anti-inflammatory factor interleukin-10 (IL-10) in hepatic macrophages and inhibiting infiltration of pro-inflammatory cells to alleviate liver damage (55). Cav-1 can also alleviate intestinal fibrosis by regulating autophagy. Decreased Cav-1 expression has been observed in both stenotic colonic tissues of Crohn’s disease patients and fibrotic intestinal tissues of dextran sodium sulfate-induced mice. Overexpression of Cav-1 can inhibit autophagy by increasing the expression of SQSTM1/p62, and then suppress the activation of fibroblasts, thereby alleviating Crohn’s disease-induced intestinal fibrosis (56).

**Figure 4:**
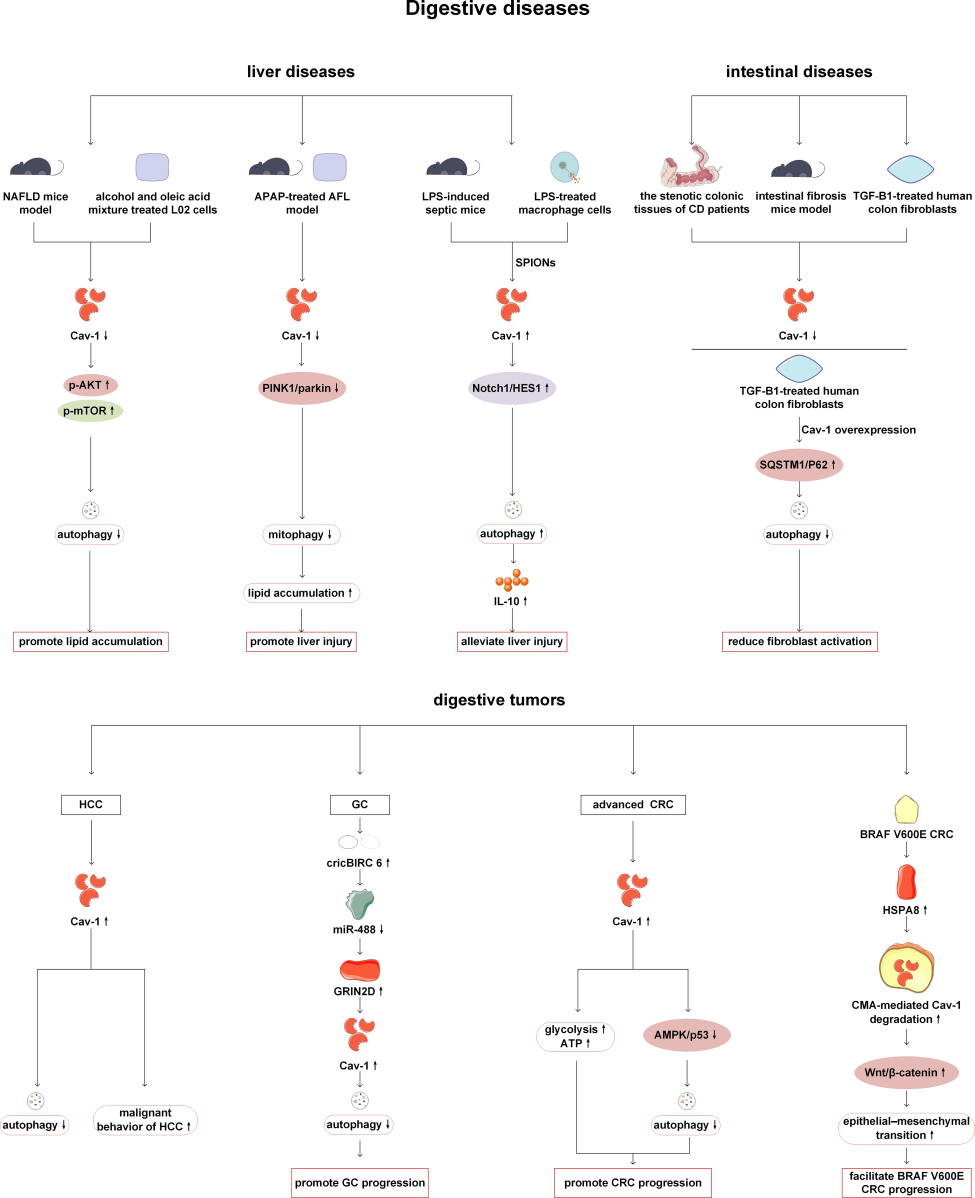
Cav-1 and autophagy in digestive diseases. In the NAFLD, Cav-1 knockdown has been shown to aggravate lipid deposition by activating the AKT/mTOR pathway and inhibiting autophagy. In an acetaminophen-aggravated alcoholic fatty liver disease model, Cav-1 knockdown inhibits PINK1/parkin-mediated mitophagy, leading to increased lipid accumulation and exacerbated liver injury. SPIONs can enhance autophagy and alleviate LPS-induced liver injury through activation of the Cav-1/Notch1/HES1 pathway. In intestinal fibrosis, overexpression of Cav-1 inhibits autophagy and reduces fibroblast activation by promoting SQSTM1/p62 accumulation. In digestive tumors, Cav-1 inhibits HCC autophagy and promotes HCC proliferation and metastasis. Specifically, in gastric cancer, the circBIRC6/miR-488/GRIN2D axis upregulates Cav-1 expression, which subsequently inhibits autophagy and promotes tumor progression. In advanced CRC, Cav-1 promotes disease progression by inhibiting the AMPK/autophagy pathway and enhancing glycolysis. Notably, in BRAF V600E CRC, HSPA8 facilitates CMA-mediated degradation of Cav-1, thereby activating the Wnt/β-catenin pathway and driving disease progression. NAFLD, nonalcoholic fatty liver disease; mTOR, mechanistic target of rapamycin kinase; AKT: protein kinase B; APAP, acetaminophen; PINK1, PTEN-induced kinase 1; LPS, lipopolysaccharide; SPIONs, superparamagnetic iron oxide nanoparticles; CD, Crohn’s disease; TGF-β1, transforming growth factor-β1; SQSTM1, sequestosome 1; HCC, hepatocellular carcinoma; circBIRC 6, circRNA BIRC 6; GC, gastric cancer; GRIN2D, glutamate ionotropic receptor N-methyl-D-aspartate type subunit 2D; CRC, colorectal cancers; HSPA8, heat shock 70 kDa protein 8; CMA, chaperone-mediated autophagy.

In addition, Cav-1 can play roles in various digestive tumors by regulating autophagy. Studies on hepatocellular carcinoma (HCC) suggest that Cav-1 can inhibit autophagy, promote proliferation, migration and angiogenesis of cancer cells. The clinical data has also revealed that expression level of Cav-1 is positively correlated with the malignancy of HCC and negatively correlated with the overall survival and recurrence time of patients (57). The highly expressed circRNA BIRC 6 (circBIRC 6) in gastric cancer (GC) patients can increase Cav-1 expression by regulating the miR-488-glutamate ionotropic receptor N-methyl-D-aspartate type subunit 2D (GRIN2D) axis, thereby inhibiting autophagy and promoting GC progression (58). In the study of fibroblasts in GC patients, it has been found that low expression of Cav-1 is closely related to poor survival rate. Further Quantum dots (QDs) -based immunofluorescence history analysis has revealed a positive correlation between the expression level of Cav-1 and the expression of autophagy marker LC3. In clinical data studies, it has been also found that high Cav-1 combined with LC3B positive expression group significantly reduce the risk of death in GC patients, indicating the synergistic effect of LC3B and Cav-1. Therefore, the study of Cav-1 and LC3B in the intercellular matrix is very important in gastric cancer patients, and this is necessary to further explore the tumor microenvironment (59). Tae-Kyu Ha et al. have revealed that Cav-1 expression is typically downregulated in early-stage colorectal cancers (CRC), while upregulated in advanced CRC. Through experiments on cell lines, mice, and human tissues, it has been demonstrated that in advanced CRC, Cav-1 exerts carcinogenic effects. Cav-1 not only promotes glycolysis and ATP production, but also contributes to tumor proliferation by inhibiting the AMPK signaling pathway and p53 dependent autophagy (60, 61). In addition, research has found that Cav-1 is associated with CMA in CRC. The transcriptional upregulation of heat shock 70 kDa protein 8 (HSPA8) in BRAF V600E CRC promotes CMA dependent degradation of Cav-1 and abrogates Cav-1-mediated inhibition of the Wnt/β-catenin pathway, thereby promoting EMT and ultimately leading to the metastasis and progression of BRAF V600E CRC (62). Therefore, we should also pay attention to the crosstalk between mitocphagy/CMA and Cav-1 in addition to conducting continuous in-depth research on autophagy (**Figure 4**).

## 5 Cav-1 and autophagy in endocrine diseases

**Figure 5:**
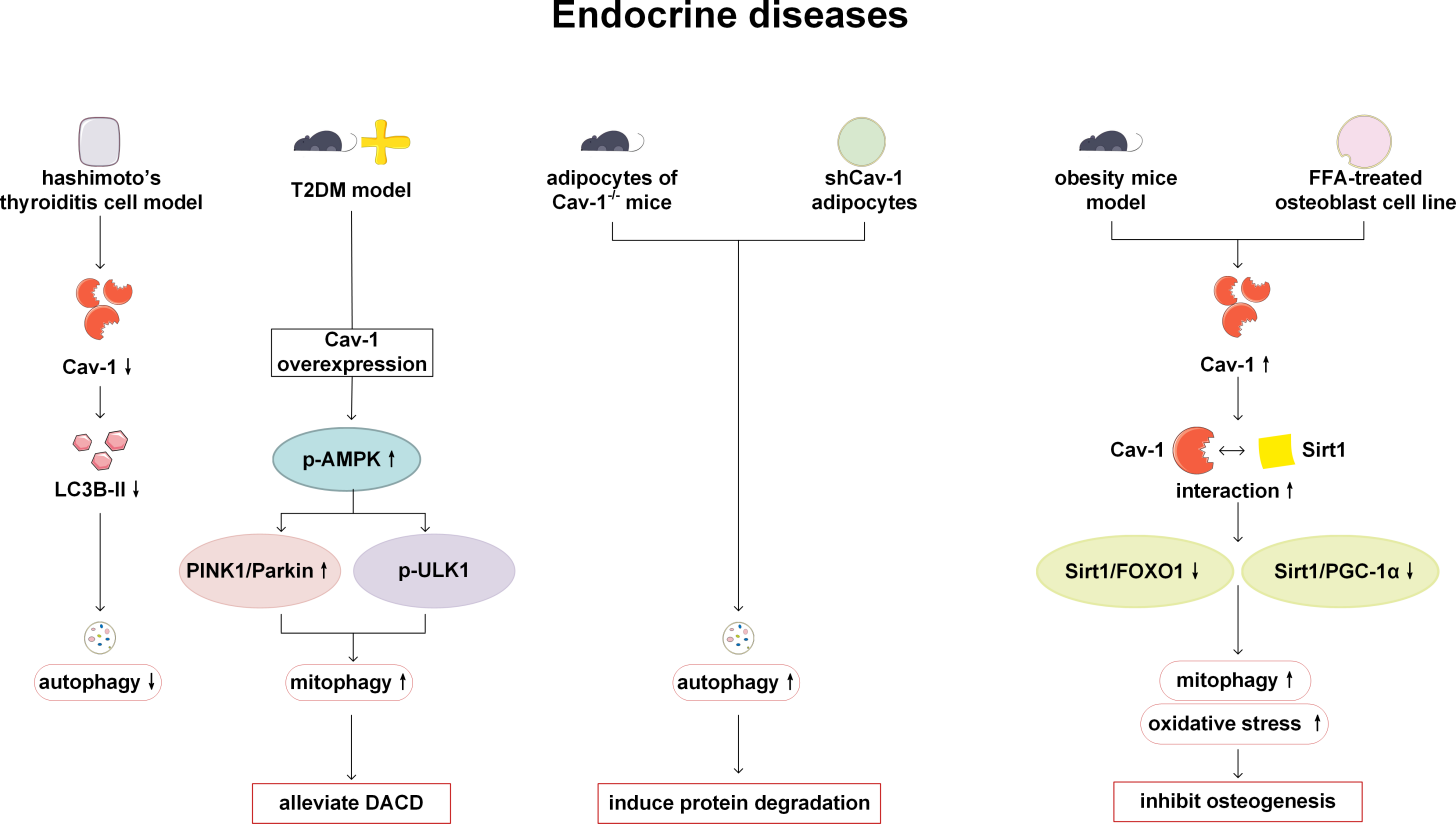
Cav-1 and autophagy in endocrine diseases. In Hashimoto’s thyroiditis, reduced expression of Cav-1 results in diminished levels of LC3B-II and suppressed autophagic activity. Overexpression of Cav-1 mitigates DACD by enhancing mitophagy via the activation of AMPK signaling pathway, and PINK1 and ULKI pathways. In Cav-1 deficient mice and humans, loss of Cav-1 induces autophagy activation and facilitates protein degradation. Conversely, overexpression of Cav-1 in obesity models inhibits bone formation by promoting mitophagy and oxidative stress through inhibition of the Sirt1/FOXO1 and Sirt1/PGC-1α pathways. LC3B, microtubule-associated protein 1 light chain 3B; T2DM, type 2 diabetes; AMPK, AMP-activated protein kinase; PINK1, PTEN-induced kinase 1; ULK1, Atg1/Unc-51 like autophagy activating kinase 1; DACD, diabetes-associated cognitive dysfunction; FOXO, Forkhead box O; Sirt1, Silent information regulator 1; PGC-1α, peroxisome proliferator-activated receptor-γ coactivator-1α.

The regulation of autophagy and Cav-1 plays a certain role in endocrine diseases such as Hashimoto’s thyroiditis, diabetes, congenital lipodystrophy and abnormal fat metabolism. Analysis of microarray data and experimental verification obtained from Cav-1 knockdown NIT-1 cell lines and mouse islets have indicated that Cav-1 can inhibit the proliferation and promote the production of pro-apoptotic cytokines. In the protein microarray, it has also been found that autophagy-related factors such as LC3A, LC3B and p62 are altered, but the change trend is inconsistent. Therefore, further research is needed on the mechanisms involved (63). Lu et al. have examined the thyroid tissue of patients with Hashimoto’s thyroiditis and have found that the expression of Th1 cytokines interleukin-1β (IL-1β) and interferon-γ (IFN-γ) is higher than that of healthy individuals, while the expression of Cav-1 is lower than that of healthy individuals. In the Hashimoto’s thyroiditis cell model treated with IL-1β and IFN-γ, the decreased expression of Cav-1 can inhibit the activity of autophagy and downregulate the expression of autophagy-related protein LC3B-II (64). This may be a new possible pathogenesis of Hashimoto’s thyroiditis. Type 2 diabetes (T2DM) can lead to the blocking of mitophagy and dysfunction of mitochondrial function, resulting in neuronal damage and diabetes-associated cognitive dysfunction (DACD). Cav-1 has been reported to enhance mitophagy and have a protective effect on DACD by activating the AMPK signaling pathway and mitophagy related pathways-Pink-1 and Atg1/Unc-51 like autophagy activating kinase 1 (ULK1) (65). Congenital generalized lipodystrophy type 4 (CGL) is caused by mutations in polymerase I and transcript release factor, also known as cavin-1 and manifested as lipoatrophy, insulin resistance, dyslipidemia and liver steatosis. In the fibroblasts of the patients, the expression of cavin1, Cav-1 and Cav-2 has been reported to be significantly lower than that of normal control subjects, and autophagy is activated (66). However, the specific role of Cav-1 has not been thoroughly studied. Abnormal lipid storage and adipodystrophy in adipose tissue has been observed in Cav-1 deficient mice and humans (9, 67). Extensive protein expression defects has been detected in Cav-1 deficient adipocytes, which are related to increased protein degradation caused by autophagy activation. Cav-1 deficiency may induce autophagy in cases of insufficient nutritional supply due to insulin resistance and fatty acid mobilization deficiencies (68). In the study of the effect of obesity on bone formation, the expression level of Cav-1 is increased in the vertebral tissue of obese mouse models and in high-fat stimulated osteoblast cell lines. The upregulated Cav-1 can promote mitophagy and oxidative stress, and inhibit osteogenesis by inhibiting the Silent information regulator 1 (Sirt 1)/Forkhead box O1 (FOXO1) and Sirt 1/peroxisome proliferator-activated receptor-γcoactivator-1α (PGC-1α) signaling pathways (69). Based on the above research, it can be seen that Cav-1 has the potential as a monitoring and therapeutic target for endocrine system diseases. However, considering the limitations of current research, more evidence support is still needed from researchers (**Figure 5**).

## 6 Cav-1 and autophagy in neurological diseases

Cav-1 and autophagy affect the permeability of the blood-brain barrier (BBB) and the function of astrocytes by acting on cerebral vascular ECs and astrocytes, thereby regulating the integrity of cerebral vascular and neural functions, and participating in the occurrence, development, and treatment of related diseases such as cerebrovascular diseases and cranial nerve dysfunction. Crucially, Cav-1 and autophagy engage in bidirectional regulation. On the one hand, Cav-1 as an autophagy inducer: Under ischemic injury or oxidative stress, Cav-1 undergoes phosphorylation at Tyr14. The phosphorylated Cav-1 binds to the BECN1/VPS34 complex and translocates into mitochondria, where it further induces autophagy, thereby alleviating cerebral infarct damage in a mouse cerebral ischemia model (70). On the other hand, autophagy can also modulate Cav-1. Autophagy is activated in astrocytes stimulated by palmitic acid (PA) and in rat hippocampal astrocytes induced by chronic high fat diet (HFD). Autophagy can induce cell apoptosis and inflammation by degrading Cav-1, while the activation of autophagy is not dependent on Cav-1 (71).

Cav-1 and autophagy can influence the progression of stroke by disrupting tight junction proteins (TJPs). The integrity of the blood-brain barrier (BBB) is highly dependent on TJPs, including Claudin-5, ZO-1, and Occludin. Previous studies have shown that in stroke, Cav-1 and autophagy are key factors in the disruption of these proteins. In brain microvascular endothelial cells (BMECs) from stroke patients, Cav-1 mediates the endocytosis and autophagic degradation of TJPs. Upregulation of Cav-1 promotes the endocytosis and cytoplasmic translocation of Claudin-5 and ZO-1 from the cell membrane (72, 73). As an upstream regulator of autophagy, Cav-1 enhances autophagic activity, as evidenced by increased colocalization with the autophagosome protein LC3B. Enhanced autophagy, in turn, drives the degradation of key TJPs (ZO-1, Occludin, Claudin-5) via the autophagy-lysosome pathway (72, 74, 75). Using an oxygen-glucose deprivation (OGD) model to mimic prolonged ischemic conditions in bEND3 cells, it has been found that increased nitric oxide (NO) triggers Cav-1 to deliver Claudin-5 to autophagosomes and promotes its degradation in an autophagy-lysosome-dependent manner (75). Ultimately, these changes lead to disruption of tight junction structure, increased BBB permeability, and exacerbation of cerebral infarction. Although Cav-1 and autophagy generally play detrimental roles during sustained ischemia/reperfusion, autophagy induced by short-term hypoxia may transiently protect the BBB and mitigate acute damage by degrading accumulated Claudin-5 and Cav-1 (72), highlighting the context-dependent role of autophagy. Post-stroke hyperglycemia during early reperfusion exacerbates BBB disruption by amplifying the Cav-1/autophagy axis. Firstly, post-stroke hyperglycemia enhances autophagic activity and increases the expression levels of Cav-1 and lysosomal markers (such as LAMP-2). Secondly, post-stroke hyperglycemia reduces ZO-1 expression through a dual mechanism (1): promoting Cav-1-mediated translocation of ZO-1 from the BMEC membrane to the cytoplasm, and (2) increasing autolysosomal degradation of ZO-1 (76). Additionally, targeting the Cav-1/autophagy axis holds therapeutic promise. Both bone marrow mesenchymal stem cell-derived extracellular vesicles (BMSC-EVs) and brain endothelial cell-derived vesicles (BEC-EVs) have been shown to significantly downregulate the elevated Cav-1 expression observed in cerebral microvessels after stroke. Dependent on the modulation of Cav-1, these EVs exert protective effects in stroke—including reduced endothelial permeability, attenuated cerebral infarction, and improved neurological function (73, 74).

**Figure 6:**
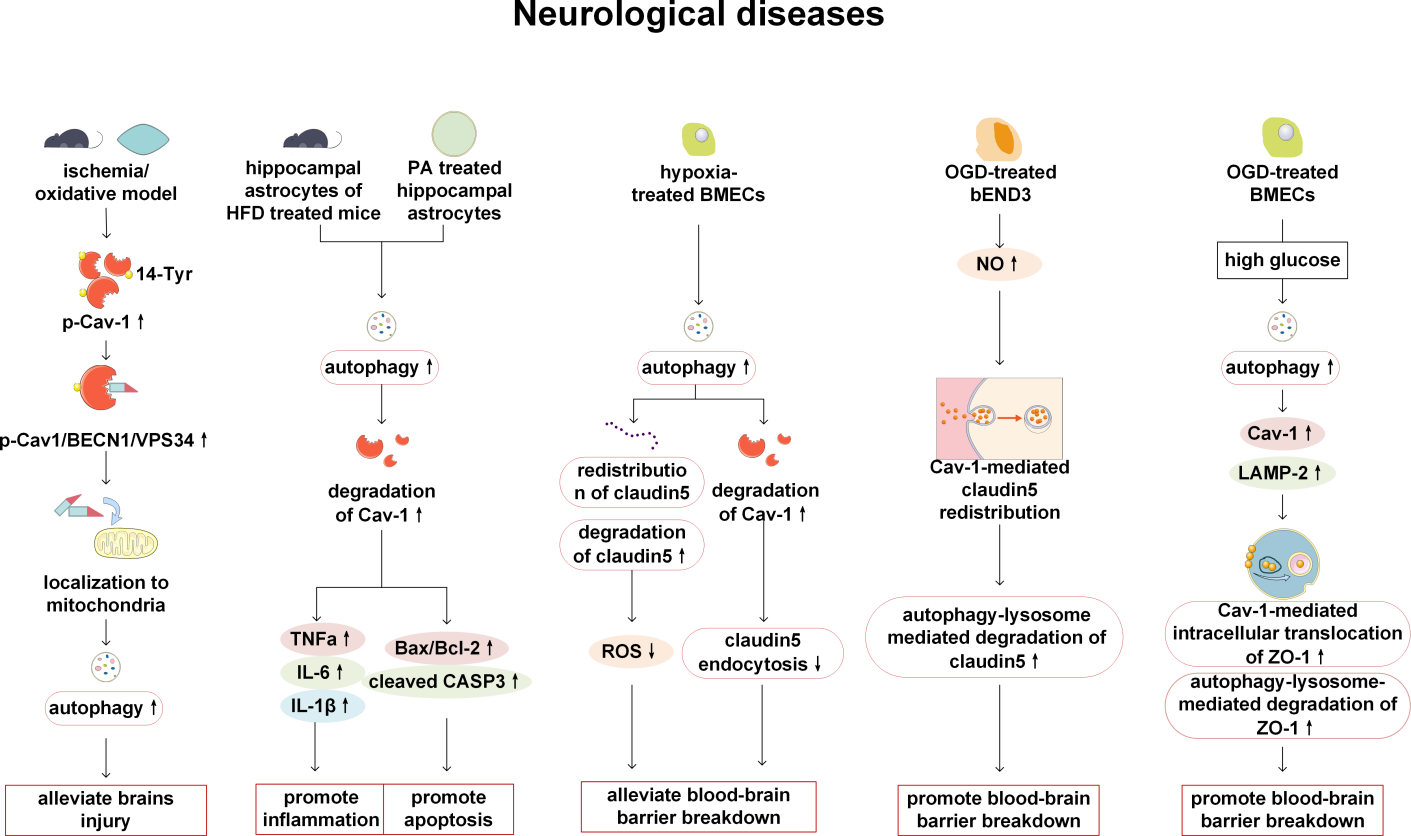
Cav-1 and autophagy in neurological diseases. p-Cav-1 interacts with the BECN1/VPS34 complex to induce autophagy, thereby alleviating brain injury in cerebral ischemia models. However, under conditions of high lipid exposure or PA stimulation, autophagy-mediated degradation of Cav-1 is enhanced, leading to astrocyte apoptosis and inflammation. In hypoxic conditions, activation of autophagy in cerebral microvascular endothelial cells mitigates hypoxia-induced blood-brain barrier dysfunction by regulating the redistribution and degradation of claudin-5 and promoting Cav-1 degradation. In the OGD model simulating ischemia, increased NO levels can promote Cav-1-mediated redistribution of claudin-5 and enhance autophagy-lysosomal-dependent degradation of claudin-5, which contributing to the disruption of the internal blood-brain barrier. Reperfusion hyperglycemia induces autophagy activation, resulting in increased Cav-1-mediated translocation and lysosomal degradation of ZO-1, thereby exacerbating brain injury. TNFα, tumor necrosis factor α; IL, interleukin; BMECs, bone Marrow Endothelial Cells; ROS, reactive oxygen species; OGD, oxygen glucose deprivation; NO, nitric oxide; LAMP, lysosomal-associated membrane protein; ZO-1, zonula occludens 1.

In summary, Cav-1 and autophagy form a tightly interconnected regulatory axis that critically governs BBB integrity and neurovascular function in stroke through their bidirectional interaction and modulation of TJPs. This multifaceted involvement positions the Cav-1–autophagy axis as a highly compelling therapeutic target for stroke intervention (**Figure 6**).

## 7 Cav-1 and autophagy in urinary diseases

**Figure 7:**
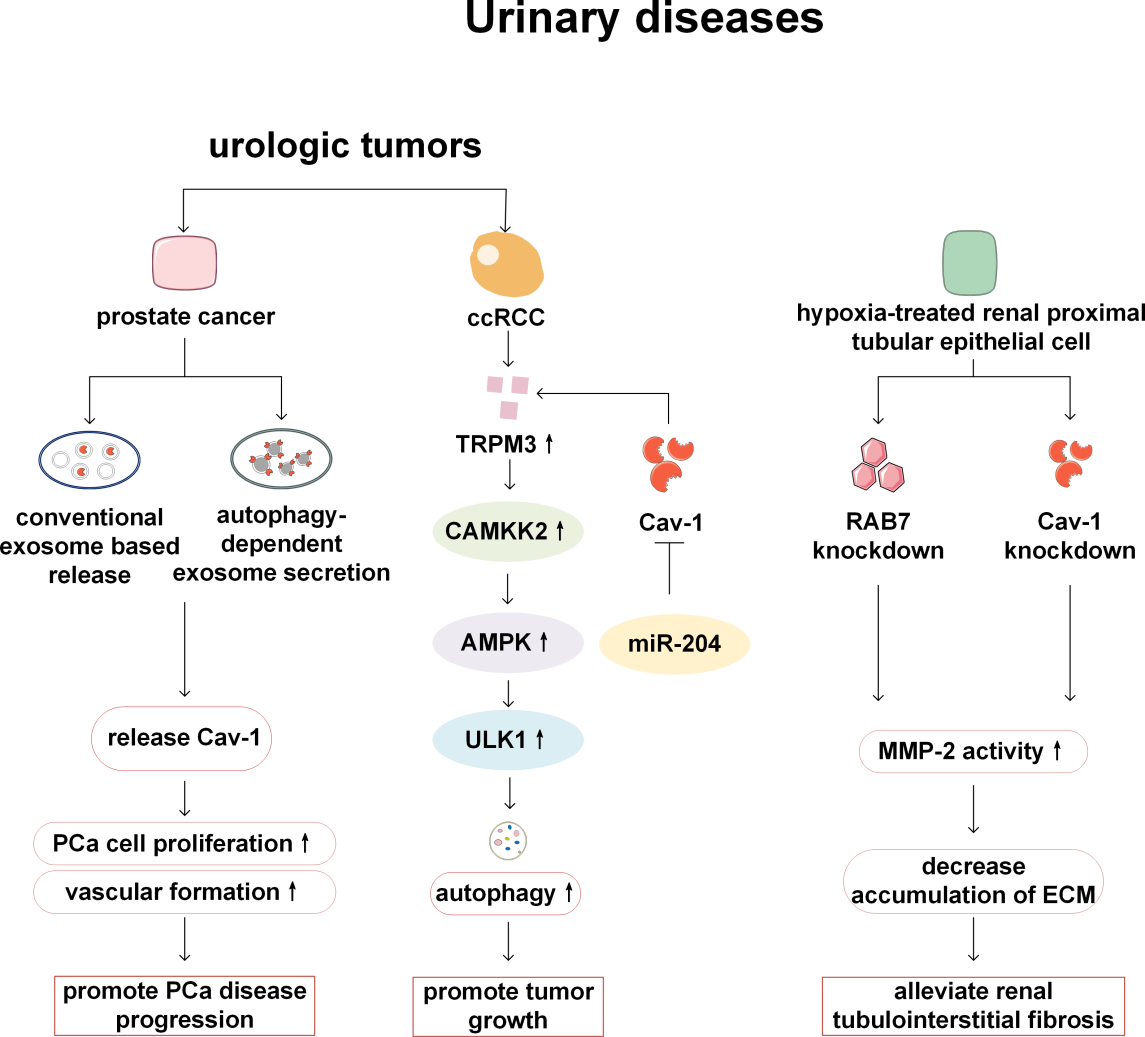
Cav-1 and autophagy in urinary diseases. In prostate cancer, Cav-1 facilitates tumor proliferation and angiogenesis via conventional exosomal secretion and autophagy-dependent pathways. In ccRCC, TRPM3 promotes tumor growth by activating the CAMKK2/AMPK/ULK1 pathway, thereby enhancing autophagy. While, the expression of TRPM3 is regulated by miR-204 and Cav-1 which is another target of miR-204. In a model of renal tubulointerstitial fibrosis, RAB7 knockdown and Cav-1 knockdown increase MMP-2 activity, thereby enhancing ECM degradation and alleviating fibrosis. PCa, prostate cancer; ccRCC, clear cell renal cell carcinoma; CAMKK2, calcium/calmodulin-dependent kinase kinase 2; AMPK, AMP-activated protein kinase; ULK1, Atg1/Unc-51 like autophagy activating kinase 1; TRPM3, Transient Receptor Potential Melastatin 3; RAB7, Ras-related protein Rab-7a; MMP-2, matrix metallopeptidase-2; ECM, extracellular matrix.

Cav-1 and autophagy play a role in the occurrence and development of urinary system diseases such as prostate cancer (PCa), clear cell renal cell carcinoma (ccRCC), tubulointerstitial fibrosis and kidney stones. Cav-1 plays a tumor-promoting role in PCa. Cav-1 secreted by PCa cells can stimulate cell proliferation and promote angiogenesis (77, 78). In the study of the mechanism of Cav-1 release, it has been found that Cav-1 can be released not only through conventional pathways within exosomes, but also through non-canonical secretory macroautophagy/autophagy pathway (79). ccRCC is a malignant tumor with early loss of von Hippel Lindau tumor suppressor protein (VHL). Research has found that the Transient Receptor Potential Melastatin 3 (TRPM3) is overexpressed in ccRCC and promotes tumor growth. Its expression is regulated by miR-204 and also influenced by Cav-1 which is another target of miR-204. TRPM3 promotes autophagy through the calcium/calmodulin-dependent kinase kinase 2 (CAMKK2)/AMPK/ULK1 pathway, providing a potential target for ccRCC therapy (80). Although Cav-1 and autophagy are involved in this study, the relationship between these two has not been further explored (80). In a hypoxia-induced tubulointerstitial fibrosis cell model, downregulating the autophagy and endocytosis regulator Ras-related protein Rab-7a (RAB7) or endocytosis regulator Cav-1 can increase the activity of matrix metallopeptidase-2 (MMP-2), promote the decomposition of extracellular matrix (ECM), and improve renal tubulointerstitial fibrosis (81). However, in this study, Cav-1 is only used as an endocytosis-related gene to prove the relationship between endocytosis and MMP-2 activity, and no further research was conducted on the relationship between Cav-1 and autophagy. In a study on calcium oxalate (CaOx) kidney stones, it is speculated that CaOx promotes kidney stone formation by downregulating the Cav-1 and low density lipoprotein receptro-related protein 6 (LRP6)/Wnt/β-catenin pathways, causing autophagy dependent ferroptosis (82). This suggests that Cav-1-autophagy may be involved in the pathogenesis of kidney stones. However, there is currently limited research on the role of Cav-1-autophagy in urinary system diseases, and still a lack of sufficient evidence to support its function. This may be a direction that researchers need to focus on in the future (**Figure 7**).

## 8 Cav-1 and autophagy in breast and reproductive diseases

Among breast related diseases, reports on Cav-1 and autophagy mainly focus on breast cancer. Autophagy enhancement and downregulation of Cav-1 expression have been observed in human breast cancer cell lines and cancer tissues, and these two changes were mutually regulated. On the one hand, autophagy activation leads to the degradation of Cav-1 in tumor cells, resulting in downregulation of Cav1 expression; On the other hand, Cav-1 deficiency can activate autophagy by promoting lysosomal function and autophagy lysosome fusion, thereby providing survival advantages for breast cancer cells, facilitating cell survival under stress conditions such as starvation, and thus promoting the development of cancer (83). It is worth noting that the regulation of autophagy by Cav-1 is phosphorylation dependent. In breast cancer cells, phosphorylated Cav-1 interacts with Mitofusin 2 (Mfn2) and Dynamin related protein 1 (Drp1), keeping them away from mitochondria, thereby reducing mitophagy. The reduction or dephosphorylation of Cav-1 can release Mfn2 and Drp1, promote mitochondrial dynamics and mitophagy, and reduce the accumulation of damaged mitochondria, thereby enabling cancer cell survival and affecting cancer treatment (84). In addition, phosphorylated Cav-1 also can reduce basal mitophagy by regulating the Rho-associated kinase (ROCK) signaling pathway and inhibiting the activation of AMPK, thereby affecting the function of mitochondria and increasing the production of reactive oxygen species (ROS), leading to further tumor progression (85). However, some studies have found that in breast cancer, the regulatory relationship between Cav-1 and autophagy may be positive. 17β-estradiol (E2) can induce tumor cell growth by promoting the activation of the high mobility group box 1 protein (HMGB1)/Cav-1 pathway, enhancing autophagy and inhibiting apoptosis in BT474 human breast cancer cells. Knockdown of Cav-1 can reduce the expression of HMGB1 and autophagy markers (LC3-II and Atg12/5) in BT474 cells, and inhibit autophagosome formation (86). Wang et al. have revealed that the anticancer drug Sanguisorba officinalis L (SA) plays an anti-tumor metastasis role in breast cancer by inhibiting hypoxia inducible factor-1α (Hif-1 α)/Cav-1 signaling, thereby affecting lysosomal function and inhibiting late stage of autophagy (87).

In addition to tumor tissue itself, tumor microenvironment is also the key to affect the invasion and metastasis of breast cancer. Cancer cells recruit supportive stromal cells from nearby endogenous tissue to promote tumor formation, making them an important component of the tumor microenvironment. When hypoxia or oxidative stress occurs during tumor growth, autophagy of the tumor matrix is activated, providing nutrients for tumor cells and reshaping the tumor microenvironment. Autophagy activation leads to the degradation of Cav-1 in tumor stromal cells (88, 89). CAFs are the main components of the tumor matrix. Breast cancer cells trigger oxidative stress in CAFs and activate two autophagic driving factors, namely HIF-1 α and NF- κ B, leading to autophagic degradation of Cav-1 in CAFs. The absence of matrix Cav-1 exacerbates oxidative stress and further promotes autophagy and mitophagy. The substances produced by matrix autophagy can be directly utilized by cancer cells to maintain growth and cellular vitality, and to combat apoptosis (90). Previous studies have also revealed that loss of stromal Cav-1 significantly increase autophagy in fibroblasts accompanied with increasing secretion of glutamine and other aminoacids (90, 91). In addition, in the co-culture model of immortalized human fibroblasts and MCF7 human breast cancer cells established by Martinez Outschool et al., glutamine, as an essential substance for cancer metabolism, can cause the low expression of Cav-1 and increase of autophagy in fibroblasts under the stimulation of high glutamine (92). Meanwhile, multiple studies have shown that stromal cells lacking Cav-1 are more prone to ROS production, with more obvious inflammatory cell infiltration, including lymphocytes, macrophages, and mast cells, and ROS can lead to mitochondrial dysfunction and DNA damage, drive autophagy and activation of fibroblasts, accelerate glucose metabolism, thereby preventing adjacent epithelial cancer cell death, providing circulating nutrients for cancer cell metabolism, and promoting cancer cell growth (88, 90, 91, 93–97). Therefore, Cav-1 is associated with an increased risk of early tumor recurrence, metastasis, and reduced overall survival, and is a matrix marker for poor prognosis. In addition, chloroquine or other autophagy/lysosomal inhibitors may also be useful anticancer drugs (98).

**Figure 8:**
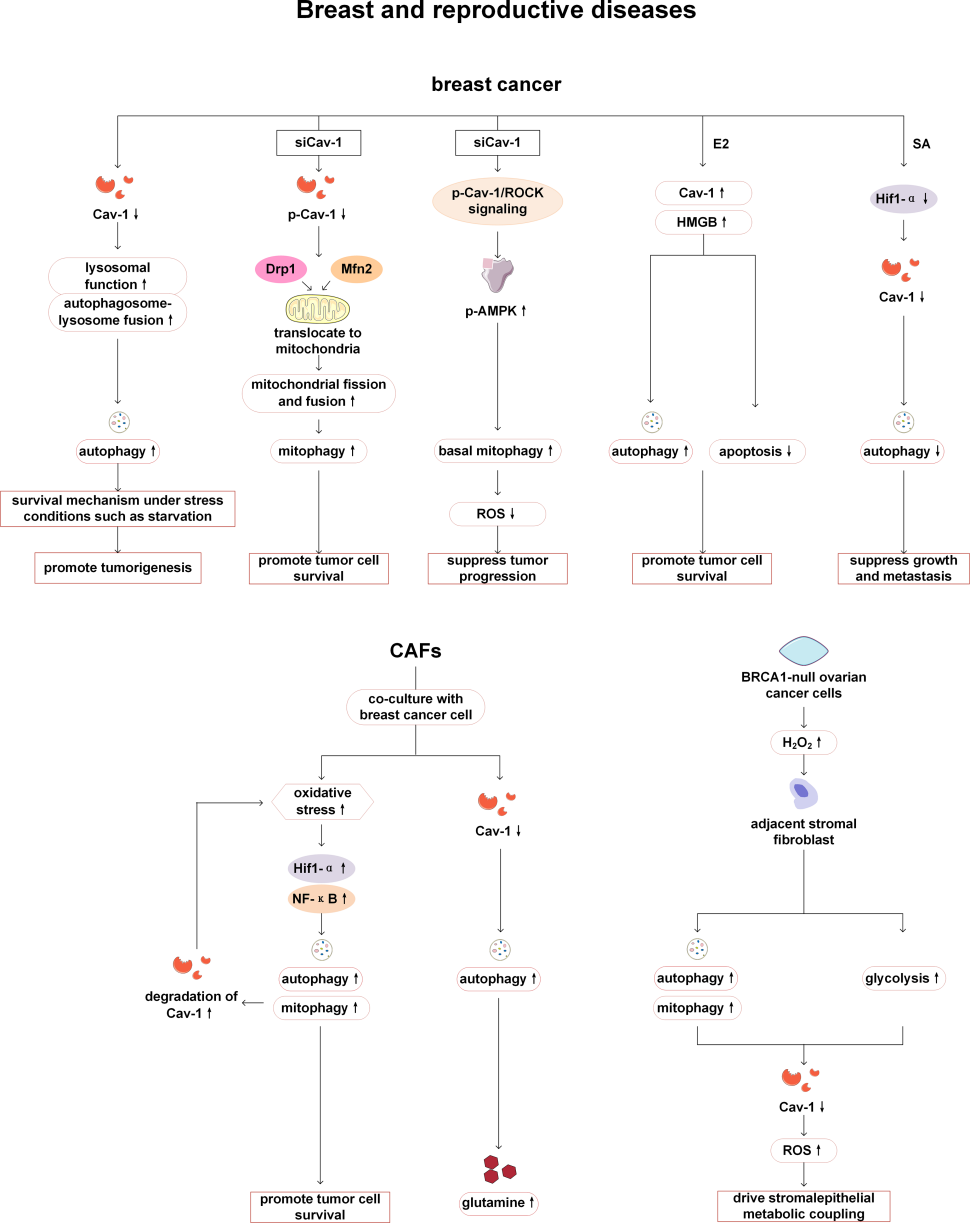
Cav-1 and autophagy in breast and reproductive diseases. In breast cancer, the downregulation of Cav-1 enhances tumor cell survival by facilitating lysosomal-autophagy fusion. The reduction in p-Cav-1 influences tumor progression by promoting mitophagy via interactions with Mfn2 and Drp1, or by activating autophagy through the ROCK/AMPK signaling pathways. E2 can stimulate tumor cell growth by enhancing the activation of the HMGB1/Cav-1 pathway, thereby promoting autophagy and inhibiting apoptosis. SA exerts an antitumor effect in breast cancer by suppressing Hif-1α/Cav-1 signaling, which subsequently inhibits autophagy. When CAFs are co-cultured with breast cancer cells, increased oxidative stress induces autophagy in CAFs and accelerates Cav-1 degradation, thereby promoting tumor cell survival. The decline in Cav-1 levels can also enhance glutamine secretion and remodel the metabolic microenvironment. In ovarian cancer, BRCA1 mutations can activate stromal autophagy and promote Cav-1 degradation, which driving metabolic coupling between stromal and epithelial cells. Mfn2, Mitofusin 2; Drp1, Dynamin related protein 1; ROCK1, Rho-associated coiled-coil kinase 1; AMPK, AMP-activated protein kinase; ROS, reactive oxygen species; E2, 17β-estradiol; HMGB1, high mobility group box 1 protein; SA, Sanguisorba officinalis L; Hif-1 α, hypoxia inducible factor-1α; CAFs, cancer-associated fibroblasts; NF-κ B, nuclear factor kappa B.

Cav-1 and autophagy have also been found to be associated with reproductive system diseases. Zeng et al. have demonstrated that low expression of Cav-1 and high expression of the autophagy marker ATG4C independently indicate reduced overall survival in epithelial ovarian cancer patients, and a positive correlation between Cav-1 and ATG4C protein expression in cancer cells and stromal cells is confirmed, so Cav-1 and ATG4C may serve as monitoring targets or signaling molecules for tumor progression. However, only statistical analysis of clinical data and immunohistochemistry analysis have been performed in this study, and no in-depth mechanism research is conducted on the relationship between Cav-1 and autophagy (99). Mutations in the breast cancer type 1 susceptibility protein (BRCA1) tumor suppressor gene are typically present in hereditary ovarian cancer, leading to the production of large amounts of hydrogen peroxide in cancer cells, which activates NF-κB, induces autophagy, mitophagy, and glycolysis processes in adjacent stromal fibroblasts, and results in Cav-1 loss caused by autophagic digestion. The cascade effect of ROS can amplify the loss of Cav-1 in surrounding stromal fibroblasts and propagate oxidative stress (100). This indicates that Cav-1-autophagy is involved in the pathogenesis of hereditary ovarian cancer (**Figure 8**).

## 9 Cav-1 and autophagy in blood diseases

**Figure 9:**
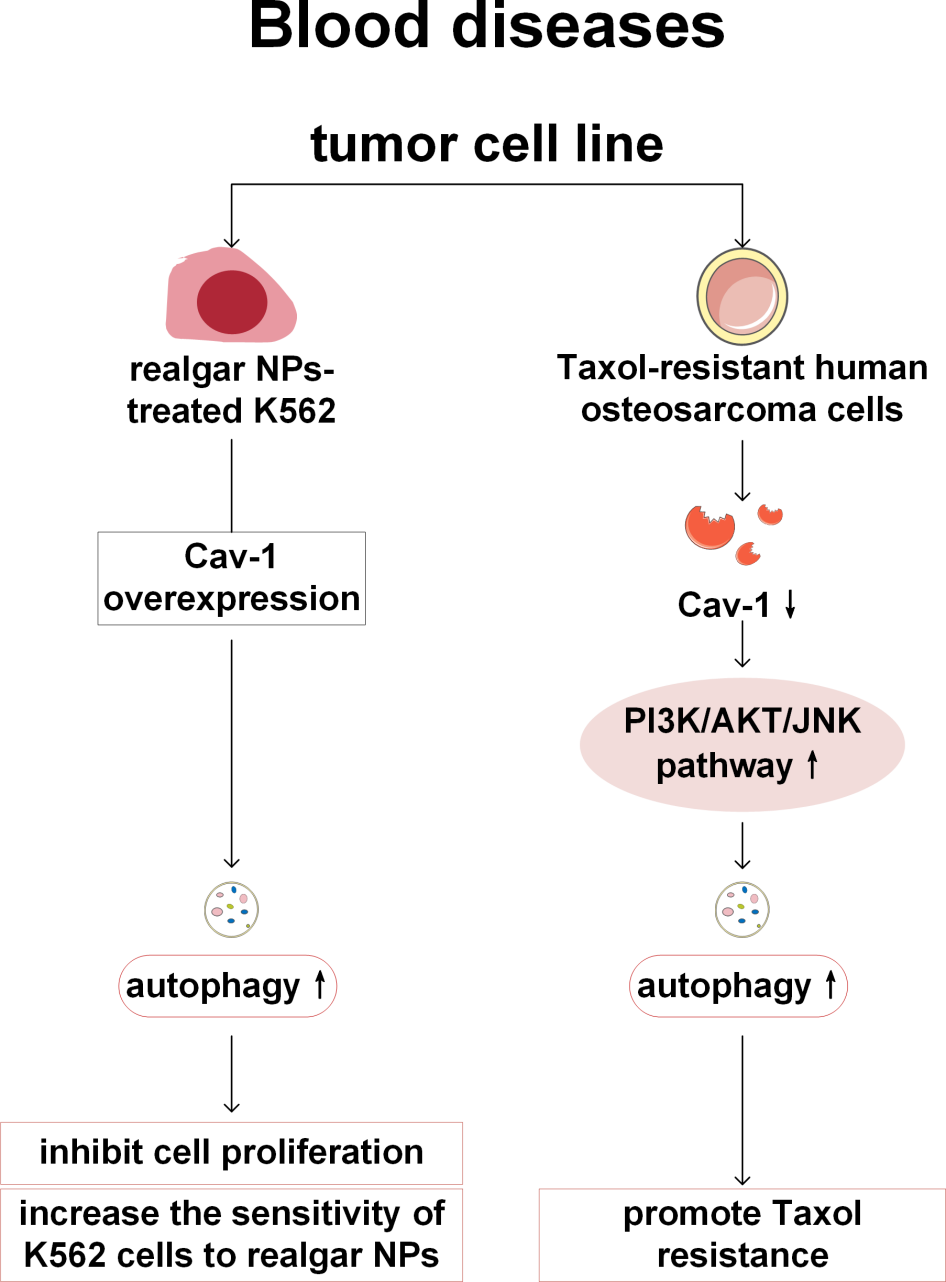
Cav-1 and autophagy in blood diseases. When realgar NPs act on CML cells, the overexpression of Cav-1 can enhance autophagy activity, increase the sensitivity of cancer cells to treatment, and inhibit cell proliferation. In the context of osteosarcoma resistance mechanisms, the expression of Cav-1 is downregulated in taxol-resistant human osteosarcoma cells, while the autophagy level is elevated via activation of the PI3K/AKT/JNK signaling pathway, thereby promoting drug resistance. NPs, Realgar nano-particles; PI3K, phosphoinositide 3-kinase; AKT: protein kinase B; JNK, c-Jun N-terminal kinases.

So far, the research on Cav-1 and autophagy in diseases of blood system is still very limited, and the relevant content is only mentioned in two articles on chronic myeloid leukemia (CML) and osteosarcoma. CML is a malignant myeloproliferative tumor characterized by the presence of the Philadelphia chromosome caused by the reciprocal translocation t (9, 22) (q34; q11) between breakpoint cluster region (Bcr) and Abelson murine leukemia (Abl) genes, resulting in the formation of the Bcr-Abl fusion gene. Shi et al. (94) have found that Realgar nano-particles (NPs) inhibit the proliferation of human CML K562 cells and stimulate the Bcr-Abl degradation to play an anticancer role, which may be related to activating autophagy, apoptosis, and promoting cell differentiation. It has also been found that overexpression of Cav-1 can be helpful for the treatment of CML by realgar NPs by activating autophagy, decreasing the proliferation of CML cells and increasing the sensitivity of K562 cells to realgar NPs (101). Therefore, Cav-1 may be beneficial for the treatment of CML, but more evidence is needed to support this. The expression of Cav-1 is related to the drug resistance of human osteosarcoma. In the Taxol-resistant osteosarcoma cells, Cav-1 is downregulated and autophagy is activated. Overexpression of Cav-1 can inhibit autophagy by attenuating the phosphoinositide 3-kinase (PI3K)-AKT-c-Jun N-terminal kinases (JNK) signaling pathway, thereby restoring the sensitivity of drug-resistant cells to Taxol (102). But whether Cav-1 can achieve clinical translation in drug-resistant patients with osteosarcoma still requires more preclinical experimental support (**Figure 9**).

## 10 Cav-1 and autophagy in other diseases

**Figure 10:**
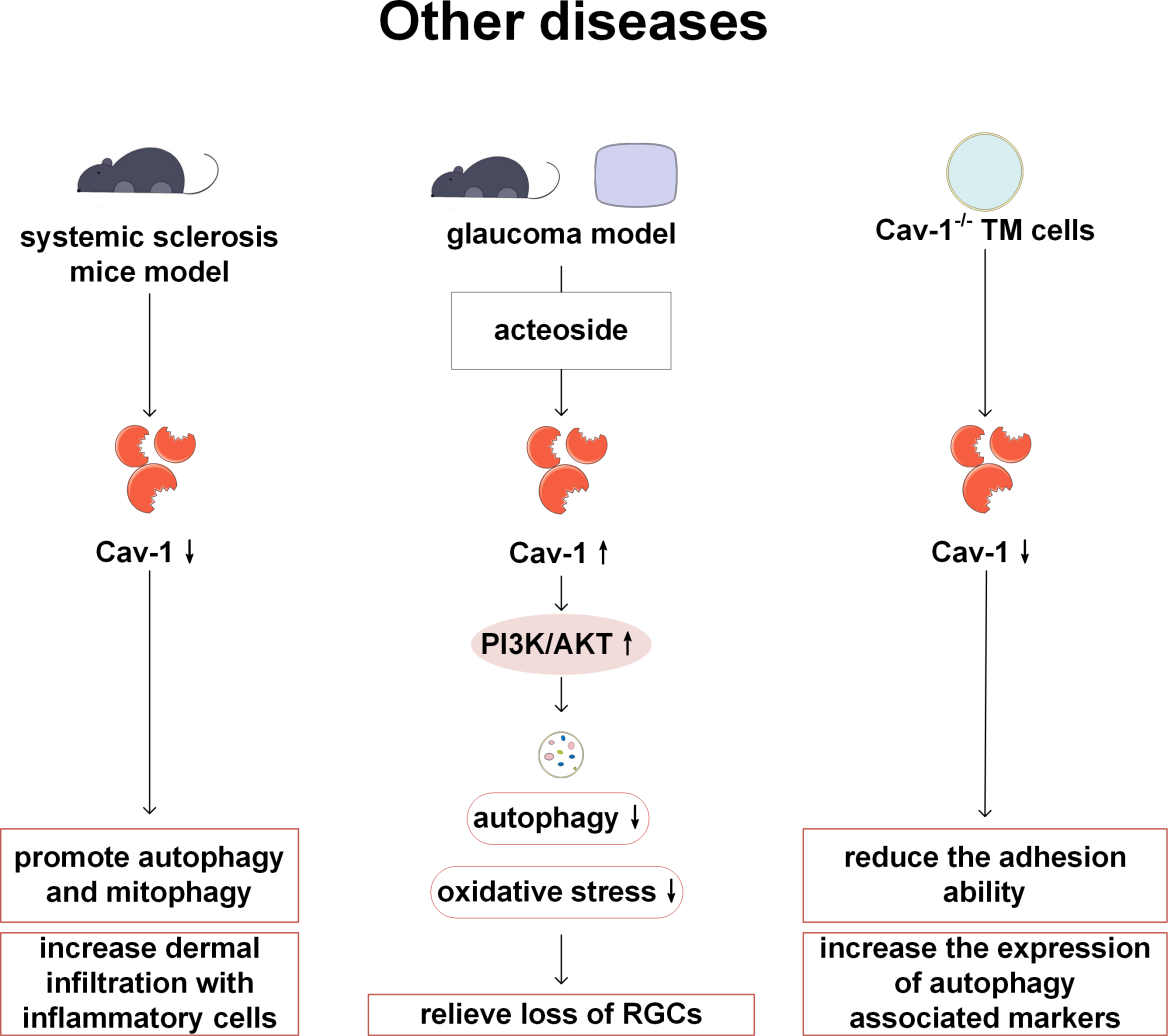
Cav-1 and autophagy in other diseases. In SSc, the low expression of Cav-1 can activate autophagy/mitophagy and enhance the infiltration of inflammatory cells. In the glaucoma model, acteoside inhibits autophagy and protects RGC by up-regulating Cav-1 and activating PI3K/AKT signaling pathway. Adhesion ability is decreased and autophagy markers are increased in Cav-1 deficient TM cells. PI3K, phosphoinositide 3-kinase; AKT: protein kinase B; RGC, retinal ganglion cell.

In addition to participating in the above-mentioned systemic diseases, Cav-1 is also related to the pathogenesis of other diseases. Cav-1 is involved in the pathogenesis of systemic sclerosis (SSc). In Castello Cros et al.’s study, it has been found that Cav-1 expression is lower in affected tissues of SSc patients compared with normal tissues, and restoration of Cav-1 expression *in vitro* experiments alleviates skin fibrosis levels. In the Cav-1^-/-^ mouse model, not only autophagy and mitophagy are activated, but also inflammatory cell infiltration is enhanced, and energy metabolism shifts towards glycolysis. Therefore, Cav-1^-/-^ mice may be used as a new preclinical model of SSc, and the use of autophagy inhibitors may also become a key therapeutic step in SSc (103). Xi et al. have found that acteoside can activate PI3K/AKT signals by upregulating Cav-1, thereby reducing autophagy and the loss of retinal ganglion cells, and exerting neuroprotective effects. Therefore, acteoside has been considered as a drug for treating glaucoma. This study has confirmed the importance of Cav-1 and autophagy in the treatment of glaucoma with acteoside by altering Cav-1 expression and using autophagy inhibitors (104). Trabecular meshwork (TM) is an important structure for maintaining intraocular pressure, and its damage is an important mechanism leading to primary open-angle glaucoma. In previous studies, lack of Cav-1 in TM cells has showed decreased adhesion and increased level of autophagy markers (ATG7, BECN1, LC3B-II). This study suggests an important regulating role of Cav-1 on the TM, however, it is still unclear how Cav-1 mediates autophagy, and further research is required in the future (105) (**Figure 10**).

## 11 Conclusion

**Figure 11:**
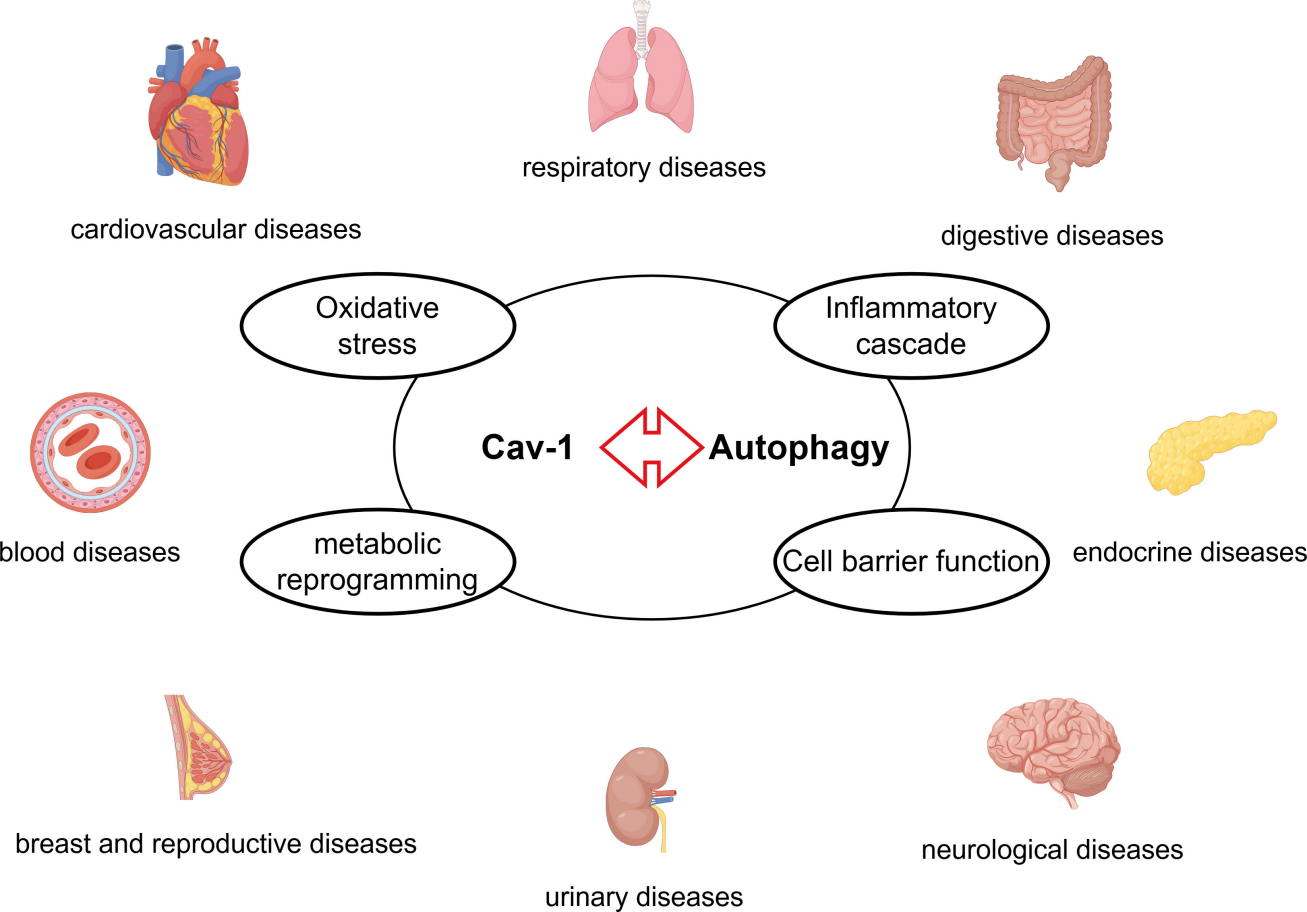
The Cav-1-autophagy interaction modulates the pathogenesis of diseases across multiple organ systems – including cardiovascular, respiratory, digestive, endocrine, nervous, urinary, breast and reproductive, and blood through common mechanisms such as oxidative stress, inflammatory cascade, metabolic reprogramming, and cell barrier function.

This review systematically explores the complex interactions between Cav-1 and autophagy in cardiovascular, respiratory, digestive, endocrine, nervous, urinary, breast and reproductive, blood systems, and other diseases (such as systemic sclerosis and glaucoma) (**Figure 11**). Cav-1 and autophagy form a bidirectional interaction network in multiple diseases. Cav-1 can positively or negatively regulate autophagy, influencing different stages of the autophagic process (induction, formation, and degradation). Conversely, autophagy regulates the degradation and localization of Cav-1. The effects of Cav-1-autophagy interactions are highly context-dependent. In various tumor cells (e.g., HCC and GC), Cav-1 often inhibits autophagy, promoting malignant progression and correlating with poor prognosis. Conversely, in the tumor stroma (particularly in CAFs), autophagic degradation of Cav-1 promotes tumor growth and metastasis by providing nutrients and metabolic support. In cardiovascular diseases, Cav-1 deficiency often activates autophagy. The activated autophagy may exert protective effects in atherosclerosis by mitigating inflammation, but can be detrimental in cardiac hypertrophy and dysfunction. The common pathological mechanisms underlying Cav-1-autophagy interactions include oxidative stress, inflammatory cascades, and metabolic reprogramming, leading to common pathological phenotypes such as endothelial dysfunction, fibrosis, and barrier disruption. Key signaling pathways involved include AMPK/mTOR, PI3K/Akt, and NF-κB. Current research demonstrates the therapeutic potential of the Cav-1/autophagy axis (possible therapeutic targets are shown in **Table 1**). For example, CSP7 ameliorates pulmonary fibrosis; hydroxychloroquine inhibits breast cancer; AAV9-mediated Cav-1 delivery rescues neuronal function in diabetes. Modulating the Cav-1/autophagy axis (e.g. inhibiting Cav-1 to enhance radiotherapy sensitivity in NSCLC, or overexpressing Cav-1 to inhibit autophagy and reverse paclitaxel resistance in osteosarcoma) may represent novel strategies to overcome therapeutic resistance in tumors. However, current research has limitations, and future studies should focus on the following areas: Besides macroautophagy, further investigation into the crosstalk between Cav-1 and mitophagy or CMA is needed especially in cancer and metabolic diseases. Evidence for the role of Cav-1/autophagy in urinary system diseases (e.g., nephrolithiasis, renal fibrosis) and hematopoietic system diseases remains limited and requires more exploration. How Cav-1 post-translational modifications determine its interaction with autophagy-related complexes and functional consequences needs elucidation. In summary, the complex interplay between Cav-1 and autophagy serves as a central hub for maintaining cellular homeostasis and responding to stress. Its dysregulation impacts numerous human diseases (including cancer, cardiovascular diseases, metabolic diseases, neurodegenerative diseases, fibrosis, etc.). A deeper understanding of its tissue-specific and context-dependent regulatory mechanisms will not only provide new biomarkers for disease diagnosis but also lay a crucial foundation for developing novel therapeutic strategies targeting this pathway.

**Table 1:** Therapeutic targets of Cav-1-autophagy axis across diseases.

Disease system	Specific disease	Therapeutic targets	Potential treatment
Cardiovascular	Atherosclerosis	Cav-1/miR-7-5p/SQSTM1	miR-7-5p inhibitors
	Diabetic Cardiomyopathy	Cav-1-Sirt6-LC3B axis	Sirt6 activators
Respiratory	NSCLC Radioresistance	Cav-1/IRGM-autophagy	Cav-1 siRNA and radiotherapy
	Pulmonary Fibrosis	Cav-1-derived 7-mer peptide	CSP7 peptide delivery
Digestive	NAFLD	Cav-1-Akt/mTOR	Cav-1 gene therapy
	HCC Progression	Cav-1-Wnt/β-catenin	Cav-1 shRNA and autophagy inducers
	BRAF V600E CRC	HSPA8-CMA/Cav-1	HSPA8 inhibitors
Endocrine	DACD	Neuron-targeted Cav-1	Cav-1 intracranial targeted therapy
	Obesity-related Osteoporosis	Cav-1-Sirt1/FOXO1	Cav-1 gene therapy
Neurological	Post-stroke hyperglycemia	Cav-1/ZO-1-autophagy	Hyperglycemia control and autophagy inhibitors
Urinary	Cisplatin Resistance	Cav-1/ROCK1/Parkin	ROCK1 inhibitors
Breast and Reproductive	Ovarian Cancer	BRCA1/NF-κB/autophagy axis	Autophagy inhibitors
	Breast Cancer Stroma	Stromal Cav-1 degradation	Chloroquine or other autophagy/lysosomal inhibitors
Other	Systemic Sclerosis	Stromal Cav-1 deficiency	Autophagy inhibitors

## Glossary

A/O: alcohol and oleic acid
Abl: Abelson murine leukemia
AKT: protein kinase B
ALI: acute lung injury
AMPK: adenosine 5’-monophosphate activated protein kinase (AMPK)
AMPK: AMP-activated protein kinase
APJ: apelin receptor
ATG5: autophagy-related protein 5
BBB: blood-brain barrier
Bc: breakpoint cluster region
BMDM: bone marrow-derived macrophages
BMECs: brain microvascular endothelial cells
BNIP3: BCL2 interacting protein 3
BRCA1: breast cancer type 1 susceptibility protein
CAFs: cancer-associated fibroblasts
CAMKK2: calcium/calmodulin-dependent kinase kinase 2
CaOx: calcium oxalate
Cav: caveolin
CAVIN1: caveolae associated protein 1
ccRCC: clear cell renal cell carcinoma
CDH6: cadherin-6
CGL: congenital generalized lipodystrophy
circBIRC 6: circRNA BIRC 6
CMA: chaperone-mediated autophagy
CML: chronic myeloid leukemia
COPD: chronic obstructive pulmonary disease
CRC: colorectal cancers
CS: cigarette smoke
CSP: Cav1 scaffolding domain peptide
DACD: diabetes-associated cognitive dysfunction
Drp1: Dynamin related protein 1
E2: 17β-estradiol
E2F1: E2F transcription factor 1
ECM: extracellular matrix
ECs: endothelial cells
Egr-1: Early growth response-1
EMT: epithelial mesenchymal transition
FOXO: Forkhead box O
GC: gastric cancer
GDM: gestational diabetes mellitus
Gli-1: glioma associated oncogene-1;GRIN2D, glutamate ionotropic receptor N-methyl-D-aspartate type subunit 2D
HCC: hepatocellular carcinoma
HCC: hepatocellular carcinoma;HFD, high fat diet
Hh: hedgehog
Hif-1α: hypoxia inducible factor-1α
HMGB1: high mobility group box 1 protein
HSPA8: heat shock 70 kDa protein 8
HSPs: heat shock proteins
HUVECs: human umbilical vein endothelial cells
IFN-γ: interferon-γ
IL-10: interleukin-10
IL-1β: interleukin-1β
IRGM: immunity-related GTPase family M protein
JNK: c-Jun N-terminal kinases
LAMP: lysosomal-associated membrane protein
LC3B: microtubule-associated protein 1 light chain 3B
LDL: low-density lipoprotein
LPS: lipopolysaccharide
LRP6: low density lipoprotein receptro-related protein 6;LVH, left ventricular hypertrophy
Mfn2: Mitofusin 2
MMP-2: matrix metallopeptidase-2
mTOR: mechanistic target of rapamycin
NAFLD: nonalcoholic fatty liver disease
NF-κB: nuclear factor kappa B
NO: nitric oxide
NPs: Realgar nano-particles
NSCLC: human non-small cell lung cancer
OGD: oxygen glucose deprivation
PA: palmitic acid
PAEC: pulmonary artery endothelial cells
PASMC: pulmonary artery smooth muscle cells
PCa: prostate cancer
PGC-1α: peroxisome proliferator-activated receptor-γ coactivator-1α
PH: pulmonary arterial hypertension
PI3K: phosphoinositide 3-kinase
PIK3C3: phosphatidylinositol 3-kinase catalytic subunit type 3
Pink-1: PTEN-induced kinase 1
QDs: quantum dots
RAB7: Ras-related protein Rab-7a
ROC: Rho-associated kinase
ROCK1: Rho-associated coiled-coil kinase 1
ROS: reactive oxygen species
SA: Sanguisorba officinalis L
SCLC: small cell lung cancer
Sirt1: Silent information regulator 1
Sirt6: Sirtuin 6
SPIONs: superparamagnetic iron oxide nanoparticles
SQSTM1: sequestosome 1
SS: shear stress
SSc: systemic sclerosis
T2DM: type 2 diabetes
TJPs: tight junction proteins
TM: trabecular meshwork
TRPC1: Transient receptor potential canonical channel 1
TRPM3: Transient Receptor Potential Melastatin 3
ULK1: Atg1/Unc-51 like autophagy activating kinase 1
VHL: von Hippel Lindau tumor suppressor protein
ZO-1: zonula occludens 1
